# An extra virgin olive oil diet impairs glycemic control, but mitigates high-fat diet-induced inflammation compared to coconut oil in mice

**DOI:** 10.3389/fnut.2026.1776312

**Published:** 2026-04-28

**Authors:** Lena Keller, Angela J. T. Bosch, Andy J. Y. Low, Laura Steiger, Stephan Wueest, Daniel Konrad, Daniel T. Meier, Pascale Vonaesch, Claudia Cavelti-Weder

**Affiliations:** 1 Department of Biomedicine, University of Basel, Basel, Switzerland; 2 Division of Pediatric Endocrinology and Diabetology, University Children's Hospital, University of Zurich, Zurich, Switzerland; 3 Children's Research Center, University Children's Hospital, University of Zurich, Zurich, Switzerland; 4 Zurich Center for Integrative Human Physiology, University of Zurich, Zurich, Switzerland; 5 Department of Fundamental Microbiology, University of Lausanne, Lausanne, Switzerland; 6 Department of Endocrinology, Diabetology and Clinical Nutrition, University Hospital Zurich (USZ) and University of Zurich (UZH), Zurich, Switzerland

**Keywords:** extra virgin olive oil (EVOO), glucose metabolism, insulinogenic index, metabolic inflammation, metabolic disease, obesity

## Abstract

**Background:**

Extra virgin olive oil is widely regarded as a metabolically beneficial dietary component due to its anti-inflammatory and antioxidant properties, but its effects on glucose homeostasis remain inconclusive. The aim of this study was to evaluate the impact of an extra virgin olive oil-based high-fat diet (E-HFD) compared to a macronutrient-matched coconut oil-based HFD (C-HFD) on glucose homeostasis and investigate potential underlying mechanisms, including inflammation, gut microbiota, and metabolic partitioning.

**Methods:**

Male C57BL/6N mice (5–7 weeks old) were fed E-HFD, C-HFD, or standard chow for up to 5 months. Glucose, insulin, and pyruvate tolerance tests and glucose-stimulated insulin secretion were performed. Immune cells in the perigonadal adipose tissue, colon, and liver were analyzed by flow cytometry. Metabolic partitioning was assessed by analyses of substrate utilization and lipid metabolism.

**Results:**

E-HFD-fed mice developed glucose intolerance within 1 week, accompanied by a blunted compensatory insulin response compared to C-HFD, which further deteriorated over time. While C-HFD led to increased adipose tissue and colonic inflammation compared to chow, E-HFD showed only mild or no inflammatory changes. Systemic inflammation and cecal microbiota composition were comparable between the HFD groups. Notably, we found increased hepatic lipid accumulation, upregulation of *de novo* lipogenesis genes, glycogen storage, and increased adipose tissue mass in E-HFD, but not in C-HFD mice, compared to chow after 1 week, indicative of preferential fatty acid storage. In contrast, C-HFD was characterized by increased circulating non-esterified fatty acids and beta-hydroxybutyrate levels compared to E-HFD mice, indicating enhanced fatty acid utilization.

**Conclusions:**

E-HFD induced pronounced glucose intolerance and a blunted compensatory insulin response, while causing less tissue inflammation than C-HFD compared to standard chow. Neither inflammation nor gut microbiota composition accounted for the observed metabolic impairment. Instead, the altered glucose homeostasis was associated with fatty acid storage and enhanced hepatic lipid accumulation. These findings point toward altered metabolic partitioning and suggest the involvement of a liver-pancreas axis in diet-dependent regulation of glucose homeostasis.

## 1 Introduction

Obesity, type 2 diabetes mellitus (T2D), and impaired glucose metabolism represent significant global health challenges with rising prevalence and associated complications (1, 2). Lifestyle interventions, such as dietary modifications, are essential in preventing and managing these conditions. Among various dietary factors, extra virgin olive oil (EVOO) has emerged as a promising component harboring potential beneficial health effects. EVOO, derived from the first mechanically cold pressing of olives, is a critical component of the Mediterranean diet, which has been associated with numerous beneficial effects, potentially including improved metabolic health (3, 4). However, the direct impact of EVOO intake on glucose homeostasis remains poorly understood.

A recent systematic review and meta-analysis of studies in humans with diverse population characteristics (i.e., type 2 diabetes, obesity, apparently healthy) and a wide range of EVOO doses (10–60 ml/day of EVOO, approximating 9–55 g/day) concluded that EVOO intake does not affect fasting blood glucose, insulin, Homeostatic Model Assessment for Insulin Resistance (HOMA-IR), and hemoglobin A1c (HbA1c) (5). In contrast, a meta-analysis of four cohort studies including a non-diabetic population reported that each 10 g daily increase in olive oil was associated with a 9% reduced risk of T2D, but only up to 15–20 g/day with no additional benefit at higher intakes (6). Additionally, the effects of olive oil on glycemic control were investigated in patients with established type 2 diabetes, which showed lower HbA1c and fasting plasma glucose upon increased EVOO intake (6). Moreover, a meal containing 10 g of EVOO improved postprandial glucose excursions and lipid profiles in patients with impaired fasting glucose (7). Although the reported exposure ranges overlap, Dehghani et al. (5) evaluated EVOO doses administered as an intervention in randomized controlled trials (RCTs), whereas Schwingshackl et al. (6) combined cohort studies, where olive oil intake is inferred from habitual dietary patterns (often the Mediterranean diet), and RCTs. They did not find any benefit above a daily dose of 15–20 g in the prevention of T2D risk.

Conflicting results were also obtained in rodent studies. Mice fed an EVOO high-fat diet (HFD; E-HFD) had improved insulin resistance and islet performance compared to mice on a lard-based HFD (8). However, the difference in body weights between the two dietary groups likely introduced a bias regarding the metabolic outcomes. Other studies showed no differences in glucose tolerance and HOMA-IR in rats fed margarine or E-HFD (9), both rich in long-chain fatty acids (LCFA). Similarly, mice fed butterfat or E-HFD showed comparable progression of metabolic dysfunction-associated steatotic liver disease (MASLD) and glucose tolerance (10).

In addition to potential metabolic effects, various studies have attributed anti-inflammatory properties to EVOO, specifically to its phenolic compounds (11–15). For example, bioactive phenols have been shown to modulate the NF-κB and MAPK signaling pathways in human colon cell line *in vitro* (16). Furthermore, hydroxytyrosol and its derivatives have been reported to promote colonic Nrf2-mediated antioxidant activity, leading to the upregulation of tight junction proteins like ZO-1 and occludin and thereby strengthening intestinal barrier integrity in mice (17). In murine models of intestinal inflammation, oral administration of oleuropein reduced the expression of inducible nitric oxide synthase (iNOS) and cyclooxygenase-2 (COX-2), while significantly decreasing the production of pro-inflammatory cytokines including TNF, IL-1β, and IL-6 (18). This anti-inflammatory effect is of particular interest, considering the association between metabolic diseases and chronic inflammation, primarily observed in adipose tissue (19). Recently, we discovered that HFDs containing coconut oil (C-HFD) or lard trigger an innate immune response in the gut, characterized by a shift toward pro-inflammatory macrophage subpopulations (20). We found that lard as a source of dietary fat significantly amplified the pro-inflammatory innate immune response and worsened glycemic control (20). Notably, colon-specific depletion of macrophages improved glucose tolerance, establishing a direct connection between gut inflammation and glucose intolerance. In the gut, various anti-inflammatory effects of EVOO have been described in the context of inflammatory bowel disease (11, 12, 21). However, the impact of an E-HFD on intestinal immunity in the context of obesity and type 2 diabetes is currently unknown.

Diet-induced alterations in the microbiota are also actively implicated in the disruption of gut immunity and inflammation, as evidenced by studies on fecal transplantation (22, 23). The microbial community in the gastrointestinal tract plays a critical role in influencing host metabolism, impacting energy balance, nutrient absorption, and immune response (24–26). A 10-week consumption of E-HFD led to several notable alterations in the composition of microbial communities in mice, resulting in greater diversity in gut microbiota when compared to mice on a lard-based HFD. Additionally, the richness of the microbial population displayed an inverse relationship with blood glucose levels (27). Specifically, the intake of phenolic compounds derived from olive oil augmented the *Bacteroidetes* population and decreased the *Firmicutes/Bacteroidetes* ratio, a pattern linked to atheroprotection (28). Another study demonstrated that lower levels of the anorexigenic (appetite suppressant) hormone leptin were inversely correlated with the presence of *Sutterellaceae, Marispirillum*, and *Mucilaginibacter diagenesis*, all of which were significantly more abundant in E-HFD mice (29).

Metabolic and immunological effects may be influenced by distinct dietary components and may not necessarily occur in parallel. This study used two well-defined plant-based HFDs with distinct fat compositions, E-HFD and coconut-based HFD (C-HFD), to explore the metabolic and immunological impacts and to determine whether these effects are interlinked. A deeper understanding of how different fat components affect metabolic and immunological health is essential for the development of effective dietary strategies to prevent and manage metabolic diseases and type 2 diabetes and its complications.

## 2 Methods

### 2.1 Mice

Male C57BL/6N mice, originally obtained from Charles River Laboratories (Germany; RRID: IMSR_CRL:027; http://www.criver.com/products-services/find-model/c57bl6-mouse? region=3616), were bred in-house and kept in specific pathogen-free conditions with *ad libitum* access to food and water at room temperature (22 °C) on a 12 h light/12 h dark cycle. If possible, littermate controls were used, and animals were randomized into experimental groups using stratified randomization based on initial body weight to ensure balanced group allocation (*n* = 3–8 mice per condition and experiment, see also figure legends with pooled experiments). Age at study start was 5–7 weeks. For glucose-stimulated insulin secretion (GSIS), however, islets were isolated from 8 to 9-week-old mice to ensure sufficient islet size. Mice were fed either a coconut-based HFD (50% of total kcal; #S8965-E022, D12331 mod.; ssniff Spezialdiäten GmbH, Soest, Germany), an extra virgin olive oil-based HFD (50% of total kcal #S8965-E020, D12331 mod.; Ssniff), or a control standard diet (chow, Extrudat 3436, Granovit AG, Roggwil, Switzerland; Supplementary Table 1). Due to the liquid nature of EVOO, its incorporation into pellets was limited to a maximum fat content of 50% of total kcal. Animals were monitored weekly for general health and wellbeing. This included assessment of appearance (eyes, coat/grooming, posture), behavior (activity), and body weight. Body weight was measured once per week. For this purpose, individual mice were removed from their cage and placed in a container positioned on a calibrated precision scale to obtain individual body weight. Blinding was not feasible during the dietary intervention due to the nature of the treatment. However, results were analyzed in a blinded fashion whenever possible. Specifically, the second investigator who assisted with *in vivo* procedures (GTTs, ITTs, and PTTs) was unaware of group allocation. Moreover, all *ex vivo* analyses were performed in a blinded manner. Upon sacrifice, animals and samples were assigned numerical codes, and group allocation was only disclosed after completion of data acquisition and primary analysis (e.g., microbiota analyses, blood measurements, immune cell profiling, and tissue analyses). Data were not included if misinjection occurred during metabolic assessments or if values were excluded by the outlier test. At the end of experiments mice were euthanized using CO_2_. All animal experiments were approved by the Cantonal Veterinary Office of Basel, Switzerland, and conducted in accordance with the Swiss Animal Welfare Act and Animal Welfare Ordinance. The experiments were carried out in compliance with the ARRIVE guidelines and conform to the ethical principles of the EU Directive 2010/63/EU for animal experiments. For more details see Methods 1.1 in Supplementary material.

### 2.2 Metabolic *in vivo* assessments

The metabolic state was assessed via intraperitoneal (ip.) glucose (GTT) and insulin tolerance tests (ITT) as described previously (30). Pyruvate tolerance tests (PTT) were performed in 3 h fasted mice after ip. injection of pyruvate (10 μl/g of 20% pyruvate solution in 0.9% NaCl, pH 7.4, Sigma-Aldrich, St. Louis, MO, USA) as described before (31). Treatments were performed in an alternating cage-wise manner to minimize cage effects and other environmental confounders. For more details see Methods 1.2 in Supplementary material.

### 2.3 Pancreatic islet isolation

Pancreatic islet isolation was performed as described by Steiger et al. (31).

### 2.4 Glucose-stimulated insulin secretion

Handpicked pancreatic mouse islets were used for glucose-stimulated insulin secretion, performed as described previously (32).

### 2.5 Quantitative real-time PCR

Tissues were snap-frozen, RNA isolated (#740955.250, NucleoSpin RNA kit, Macherey-Nagel GmbH & Co. KG, Düren, Germany), and cDNA synthesized (#A2801, GoScript™ Reverse Transcription Mix, Promega Corporation, Madison, WI, USA). qPCR was performed with GoTaq qPCR Master Mix (#A6002, Promega) on a ViiA7 system (Thermo Fisher Scientific, Waltham, MA, USA). Primers were ordered from Microsynth AG (Balgach, Switzerland); sequences are summarized in Supplementary Table 2. Data were normalized to *B2m, Ppi, a*nd *Gapdh*, and expressed as –ΔΔC_t_ values.

### 2.6 Immune cell isolation

#### 2.6.1 Colon

Colons were cleaned, sectioned, and epithelial cells were removed by shaking in Hank's Balanced Salt Solution (HBSS) with 2 mM EDTA (Gibco, Waltham, MA, USA). Tissues were digested using collagenase VIII (#C2139; Sigma-Aldrich, USA), homogenized, and filtered. Leukocytes were enriched using percoll gradient (40%/70%; #GE17-0891-01; GE Healthcare, Chicago, IL, USA).

#### 2.6.2 Adipose tissue

Perigonadal fat pads were minced and digested with 1.5 mg/ml collagenase IV (#C2139; Worthington Biochemical Corporation, Lakewood, NJ, USA) mix (1 × HBSS, 10 mM 4-(2-hydroxyethyl)-1-piperazineethanesulfonic acid (HEPES) (Gibco, USA), and 8.25 μg/ml DNAse I). Followed by filtration and erythrocyte lysis.

#### 2.6.3 Liver

Livers were cut into small pieces, digested with collagenase IV mix (see adipose tissue), vortexed mid-process, filtered, and immune cells were enriched using a percoll gradient as described above. For more details see Methods 1.3 in Supplementary material.

### 2.7 Flow cytometry of immune cells

Fc receptor was blocked (CD16/32) and the corresponding antibody staining mix (Supplementary Table 3) was added and incubated in the dark on ice. Cells were washed and resuspended in FACS buffer (1 × DPBS, 0.5% BSA, 5 mM EDTA) before acquiring with a BD LSR II Fortessa (BD Biosciences, San Jose, CA, USA). Data analysis was performed using FlowJo software 10.8.2 (BD Biosciences, Ashland, OR, USA). For more details and gating strategies see Methods 1.4 and 1.5 and Supplementary Figures 1–3 in Supplementary material.

### 2.8 Hematoxylin and eosin staining (H&E)

Hematoxylin-eosin (H&E) staining was performed according to established protocols, and the NAS-score was assessed in a blinded fashion (33).

### 2.9 Plasma cytokine analysis

Blood was collected at the termination of the experiment by heart puncture into Microtainer^®^ K2E tubes (BD Biosciences, Franklin Lakes, NJ, USA). Samples were kept on ice and centrifuged (12,290 × g, 5 min, 4 °C) to separate plasma. Plasma was aliquoted and stored at −80 °C until analysis. IL-1β, IL-6, and TNF were measured using the MESO SECTOR S 600 (Meso Scale Diagnostics, LLC, Rockville, MD, USA). For more details see Methods 1.3 in Supplementary Table 4.

### 2.10 Cecal sample collection, DNA extraction, and sequencing

Cecal contents were collected, frozen at −80 °C, and DNA was extracted (QIAamp PowerFecal Pro kit, Qiagen GmbH, Hilden, Germany). 16S rRNA sequencing (V4 region) was performed by Novogene, and sequences were analyzed using the DADA2 pipeline and Silva Database (version 138: Max Planck Institute for Marine Microbiology, Bremen, Germany) (34, 35). Microbiome analysis was performed within the phyloseq environment (36). Statistical analyses were performed in R. Differential abundance analysis across all assigned sequence variants (ASV) was done using DESeq2, which fits a negative binomial generalized linear model (GLM) for each taxon and with Benjamini–Hochberg correction for multiple testing. Comparisons of relative abundances were done using a Wilcoxon rank-sum test.

### 2.11 Total liver lipids

Liver lipids were extracted from homogenized tissues using a chloroform-methanol mixture and quantified by the sulfophosphovanillin method (37).

### 2.12 Liver glycogen

Liver glycogen was measured as remaining tissue glucose. Snap-frozen liver tissue was dissolved and disrupted using HCl. Glucose was determined with the Glucose Assay Reagent Kit (#G3293, Sigma-Aldrich, USA) following the manufacturer's instructions.

### 2.13 Plasma non-esterified fatty acids (NEFA)

NEFA were determined by NEFA HR(2) kit (FUJIFILM Wako Chemicals, Osaka, Japan).

### 2.14 Liver enzymes and lipids

Liver enzymes and blood lipids were measured using a Cobas^®^ 8000 modular analyser (Roche Diagnostics, Basel, Switzerland).

### 2.15 Beta-hydroxybutyrate

Beta-hydroxybutyrate was either measured using a FreeStyle Optium Neo Meter (Abbot Diabetes Care, Witney, UK) with a drop of blood from the tip of the tail (1 week of HFD) or in frozen plasma using the Thermo Scientific™ Indiko™ Plus Clinical Chemistry Analyzer (Thermo Fisher Scientific, USA; 5 months of HFD).

### 2.16 Statistical analysis

The data are presented as mean ± standard error of the mean (SEM), with the numbers (*n*) of experiments and mice indicated in the figures. An unpaired Mann-Whitney *U*-test with a two-tailed distribution was used to test the statistical difference between two groups. To test the statistical differences between three or more groups, one-way or two-way ANOVA with multiple comparisons was performed using the Tukey test for correction for multiple comparisons. Statistical analyses were done using Prism 10 software (GraphPad Software, San Diego, CA, USA). Two-sided *p*-values of 0.05 or less were considered statistically significant.

For further details, see Supplementary material.

## 3 Results

### 3.1 E-HFD leads to pronounced glucose intolerance due to blunted compensatory insulin secretion

**Figure 1:**
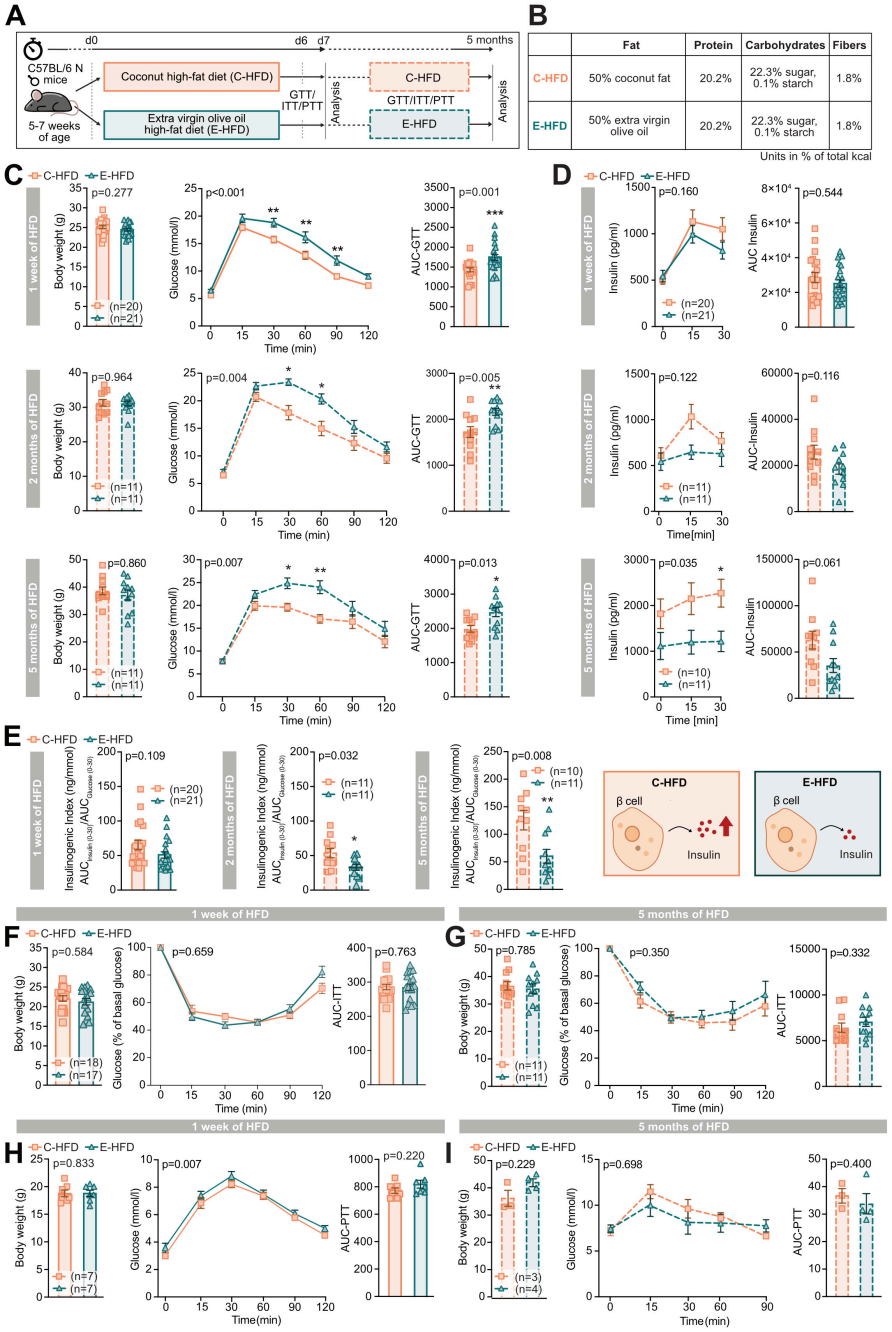
E-HFD leads to pronounced glucose intolerance due to blunted compensatory insulin secretion. 5–7-week-old wild-type (wt) C57BL/6N male mice were fed either a coconut-based HFD (C-HFD, orange squares) or extra virgin olive oil-based HFD (E-HFD, green triangles) for 1 week or up to 5 months. **(A)** Experimental setup (d = day) and **(B)** table of diet composition. **(C)** Body weight, intraperitoneal glucose tolerance test (GTT), and area under the curve (AUC) after 1 week, 2, and 5 months of HFD. **(D)** Insulin values for the first 30 min of the GTT and AUC, respectively. **(E)** Insulinogenic index after 1 week, 2, and 5 months of HFD. **(F)** Body weight, intraperitoneal insulin tolerance test (ITT), and AUC after 1 week and **(G)** 5 months of HFD. **(H)** Body weight, intraperitoneal pyruvate tolerance test (PTT), and AUC after 1 week and **(I)** 5 months of HFD. Data represent five (**C, D, E**: for 1 week of HFD), three **(F)**, two (**C, D**: for 2 and 5 months of HFD, **G**), and one **(H, I)** independent experiments, with each data point representing an individual mouse. *E-HFD vs. C-HFD; *p < 0.05; **p < 0.01; ***p < 0.001; two-way ANOVA, and multiple comparison or unpaired Mann–Whitney *U*-test with two-tailed distribution. Data are Mean ± SEM; *p* = *p*-value.

To explore whether a high fat-diet (HFD) based on EVOO exhibits distinct metabolic effects compared to coconut oil, we assessed glycemic control by intraperitoneal glucose (GTTs), pyruvate (PTTs), and insulin tolerance tests (ITTs) in mice fed either EVOO- (E-HFD) or coconut oil-based HFDs (C-HFD; Figures 1A, B). A chow group was included to contextualize the response to HFD (Supplementary Figure 4). Food intake and stomach weights were comparable in both HFD groups (Supplementary Figure 5A). We found pronounced glucose intolerance in E-HFD mice after only 1 week compared to C-HFD mice, which persisted up to 5 months of HFD feeding (Figure 1C). Importantly, body weights remained comparable between both HFD groups at all time points (Figure 1C). In both experimental conditions, insulin levels increased over time. As expected under high-fat feeding, C-HFD mice exhibited a compensatory increase in insulin secretion. In contrast, this compensatory response was blunted in E-HFD-fed mice. Specifically, insulin secretion was dampened after 5 months (at 30 min) in E-HFD-fed mice when compared to C-HFD fed mice (Figure 1D). Given the impaired glucose tolerance with the absence of an adequate compensatory insulin response observed in E-HFD mice, we calculated the insulinogenic index, which declined in E-HFD mice from 1 week to 5 months (Figure 1E). Interestingly, insulin sensitivity was not different between E- and C-HFD fed mice after 1 week or 5 months, when tested with an ITT (Figures 1F, G). Accordingly, gluconeogenesis assessed by a PTT was unchanged after 1 week or 5 months of E- and C-HFD, respectively (Figures 1H, I).

Hence, E-HFD induced pronounced glucose intolerance and a blunted compensatory insulin response, but did not affect insulin resistance within the 5-month intervention when compared to C-HFD in mice.

### 3.2 E-HFD leads to an islet-extrinsic defect in insulin secretion *in vivo*

**Figure 2:**
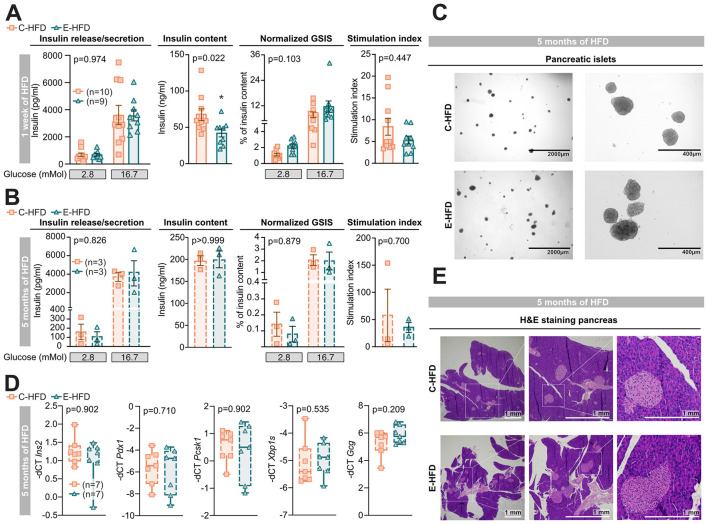
E-HFD leads to an islet-extrinsic defect in insulin secretion *in vivo*. 8–9-week-old wt C57BL/6N male mice were fed either a C-HFD (orange squares) or E-HFD (green triangles) for 1 week. **(A, B)** Glucose-stimulated insulin secretion (GSIS) of isolated pancreatic islets after 1 week **(A)** or 5 months of HFD feeding **(B)**. **(C)** Representative picture of isolated pancreatic islets after 5 months of HFD feeding. **(D)** qPCR analysis of *Ins2* (insulin 2), *Pdx1* (pancreatic and duodenal homeobox 1), *Pcsk1* (proprotein convertase subtilisin/kexin type 1), *Xbp1s* (X-box binding protein 1), and *Gcg* (glucagon) of isolated pancreatic islets after 5 months of HFD. **(E)** Representative picture of hematoxylin and eosin (H&E) staining of the pancreas after 5 months of HFD feeding. Data represent two **(A)** and one **(B–E)** independent experiments, with each data point representing an individual mouse. Two-way ANOVA, and multiple comparisons or unpaired Mann–Whitney *U*-test with two-tailed distribution. Data are Mean ± SEM; *p* = *p*-value.

To assess the underlying mechanism of the blunted compensatory insulin response upon E-HFD, we performed a glucose-stimulated insulin secretion (GSIS) assay on isolated pancreatic islets. After 1 week of HFD, basal insulin release and glucose-stimulated insulin secretion were comparable between E- and C-HFD (Figure 2A). However, a significant decrease in islet insulin content was detected in islets of E-HFD mice. When insulin secretion was normalized to the insulin content, no statistically significant difference was observed between both HFDs. Accordingly, the stimulation index was also comparable between the groups. Similarly, after 5 months of HFD feeding, the GSIS, insulin content and the stimulation index were not altered between islets isolated from E- or C-HFD fed mice (Figure 2B). Macroscopically isolated pancreatic islets showed no obvious alterations between both HFDs (Figure 2C). To investigate changes within the pancreatic islet transcriptome that might explain the *in vivo* decreased insulin secretion, qPCR of isolated pancreatic islets was performed. However, no significant differences were observed in selected prototypical genes, such as *Ins2*, maturation and development genes (*Pdx1, Pcsk1*), ER-stress gene (*Xbp1s*), or alpha-cell defining gene (*Gcg*; Figure 2D). Furthermore, no histological alterations were observed in the pancreas, particularly in the endocrine tissue, between E- and C-HFD mice when examined using hematoxylin and eosin (H&E) staining (Figure 2E).

In sum, in contrast to a pronounced insulin secretion defect *in vivo, ex vivo* pancreatic islet function was similar in E- or C-HFD fed mice, suggesting an islet-extrinsic factor that reduces beta-cell function *in vivo* in E-HFD-fed mice.

### 3.3 C-HFD, but not E-HFD, causes a M1a-driven adipose tissue inflammation and a pronounced inflammatory shift in colonic macrophages

**Figure 3:**
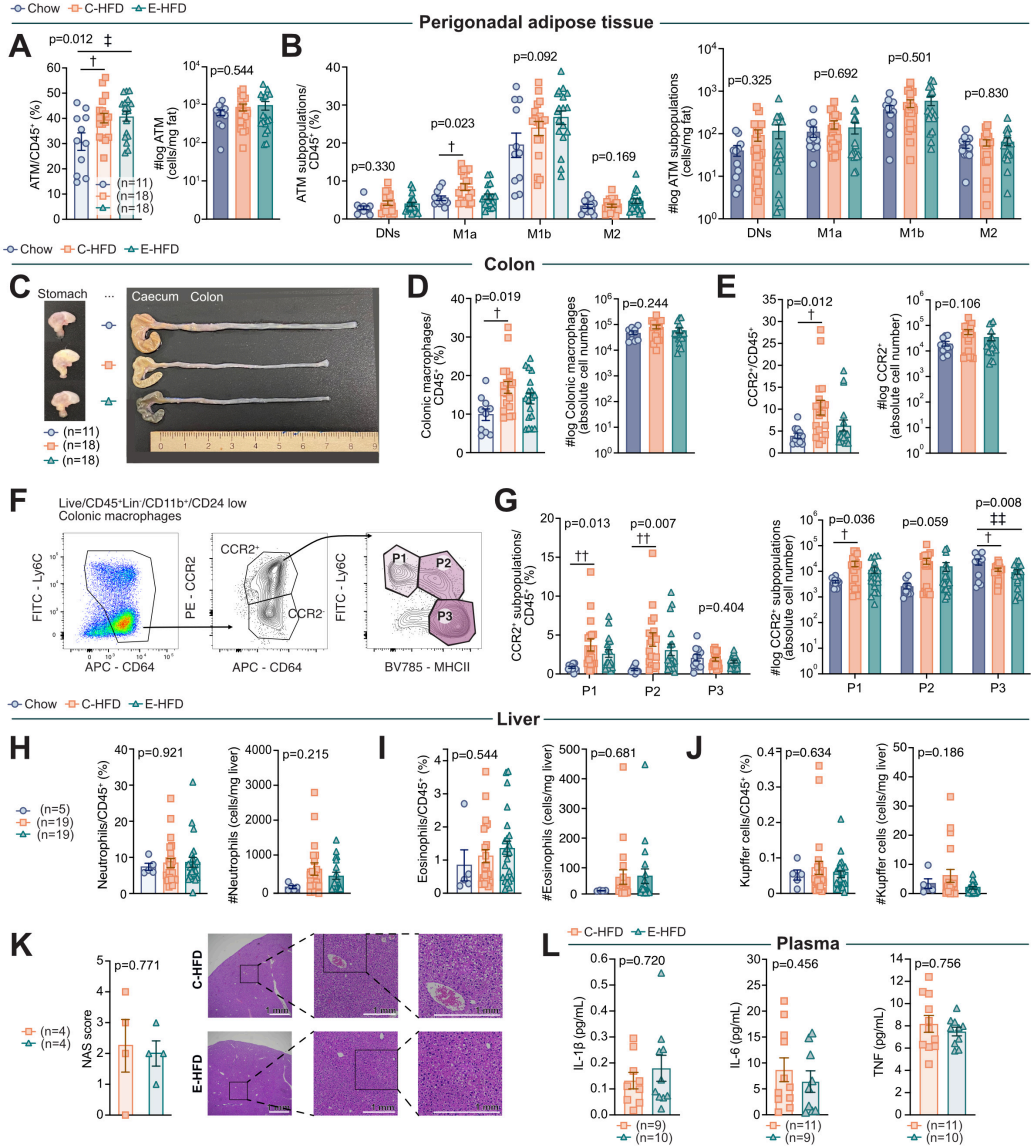
C-HFD, but not E-HFD, causes M1a-driven adipose tissue inflammation and a pronounced inflammatory shift in intestinal macrophages. 5–7-week-old wt C57BL/6N male mice were fed either a control chow diet (blue circles), a C-HFD (orange squares), or an E-HFD (green triangles) for 1 week. **(A)** Analysis of flow cytometry frequencies of perigonadal adipose tissue macrophages (ATMs) and absolute cell numbers. **(B)** Analysis of flow cytometry frequencies of ATM subpopulations and absolute cell numbers. **(C)** Representative intestines of all different treatment groups. **(D)** Analysis of flow cytometry frequencies of colon macrophages and absolute cell numbers; and **(E)** colon CCR2^+^ macrophages and absolute cell numbers. **(F)** Gating strategy of colon macrophage subpopulations. **(G)** Analysis of flow cytometry frequencies of colon CCR2^+^ macrophage subpopulations (P1–P3) and absolute cell numbers. **(H)** Analysis of flow cytometry frequencies of liver neutrophils, **(I)** liver eosinophils, **(J)** and liver Kupffer cells and absolute cell numbers, respectively. **(K)** Non-Alcoholic Steatohepatitis (NAS) score and representative H&E staining of liver sections from C- or E-HFD-fed mice. **(L)** Plasma pro-inflammatory cytokines IL-1β, IL-6, and TNF. Absolute cell numbers are shown in darker shades of the bars. Data represent four **(A, B, D, E, G)**, three **(H–J)**, two **(L)**, and one **(K)** independent experiments, with each data point representing an individual mouse. *E-HFD vs. C-HFD; ‡E-HFD vs. chow; †C-HFD vs. chow. *,‡,†*p* < 0.05; **,‡‡,††*p* < 0.01; ***,‡‡‡,†††*p* < 0.001; Ordinary one-way ANOVA and multiple comparisons; Data are Mean ± SEM; *p* = *p*-value.

To investigate whether the inflammatory response correlates with the glucose intolerance caused by E-HFD as a potential underlying mechanism, we compared immune cell populations across various metabolically important tissues in mice fed either an E- or a C-HFD compared to a control chow (gating, see Supplementary Figures 1–3). In perigonadal adipose tissue, we observed an increase in the frequency ATMs after 1 week of both HFDs, although the absolute ATM numbers remained unchanged when compared to a control chow diet (Figure 3A). Moreover, C-HFD significantly increased the frequency of the inflammatory M1a ATM subpopulation compared to a control chow (Figure 3B), while absolute cell numbers were not different.

In the gut, no morphological differences were observed between the two different HFDs (Figure 3C, Supplementary Figure 5B). We then assessed the effect of the two HFDs on colonic immune cells (gating see Supplementary Figure 2). C-HFD resulted in a significant increase in the frequency of colonic macrophages compared to chow-fed mice (Figure 3D). Moreover, C-HFD, but not E-HFD, caused a relative increase in pro-inflammatory colonic macrophages expressing *Ccr2* (containing subpopulations P1, P2 and P3, Figures 3E, F). In addition, only C-HFD resulted in a relative increase of pro-inflammatory P1 and P2 macrophages and a significant increase in the absolute number of pro-inflammatory P1 macrophages compared to control mice. Both HFDs showed significantly fewer absolute numbers of transitory P3 macrophages (Figure 3G).

When assessing the immune cell composition of the liver as an important regulator of glucose and lipid metabolism, we did not observe differences in neutrophils, eosinophils, and Kupffer cells from mice fed either a C- or E-HFD for 1 week (Figures 3H–J, gating see Supplementary Figure 3). To rule out liver damage, we analyzed plasma liver enzymes such as alkaline phosphatase, alanine aminotransferase, and aspartate aminotransferase, which showed no changes (Supplementary Figure 5C). Analysis of H&E staining revealed comparable Non-Alcoholic Steatohepatitis (NAS) scores between both HFD groups (Figure 3K).

Systemically, we assessed the circulating pro-inflammatory cytokines interleukin-1β (IL-1β), interleukin-6 (IL-6), and tumor necrosis factor (TNF). We found no differences in systemic IL-1β, IL-6, and TNF between E- and C-HFD mice after 1 week of HFD (Figure 3L). Accompanying this, there were no differences in neutrophils, eosinophils, lymphocytes, and monocytes in the blood of both HFDs compared to chow diet (Supplementary Figure 5D).

Similar to the metabolic phenotype, we assessed long-term effects of HFDs on inflammation. While adipose tissue inflammation persisted in C-HFD mice compared to control diet-fed mice, there was no inflammation in the colon or liver at that later timepoint (Supplementary Figure 6).

Thus, while C-HFD induced early adipose tissue and colonic inflammatory changes compared to chow, E-HFD-fed mice displayed impaired glucose tolerance in the absence of increased inflammation. Notably, inflammatory readouts did not differ significantly between C- and E-HFD-fed mice. These results suggest that the inflammatory parameters assessed in our study do not explain the metabolic phenotype observed in E-HFD-fed mice.

### 3.4 Decreased alpha diversity and relative abundance of given genera upon both HFDs

**Figure 4:**
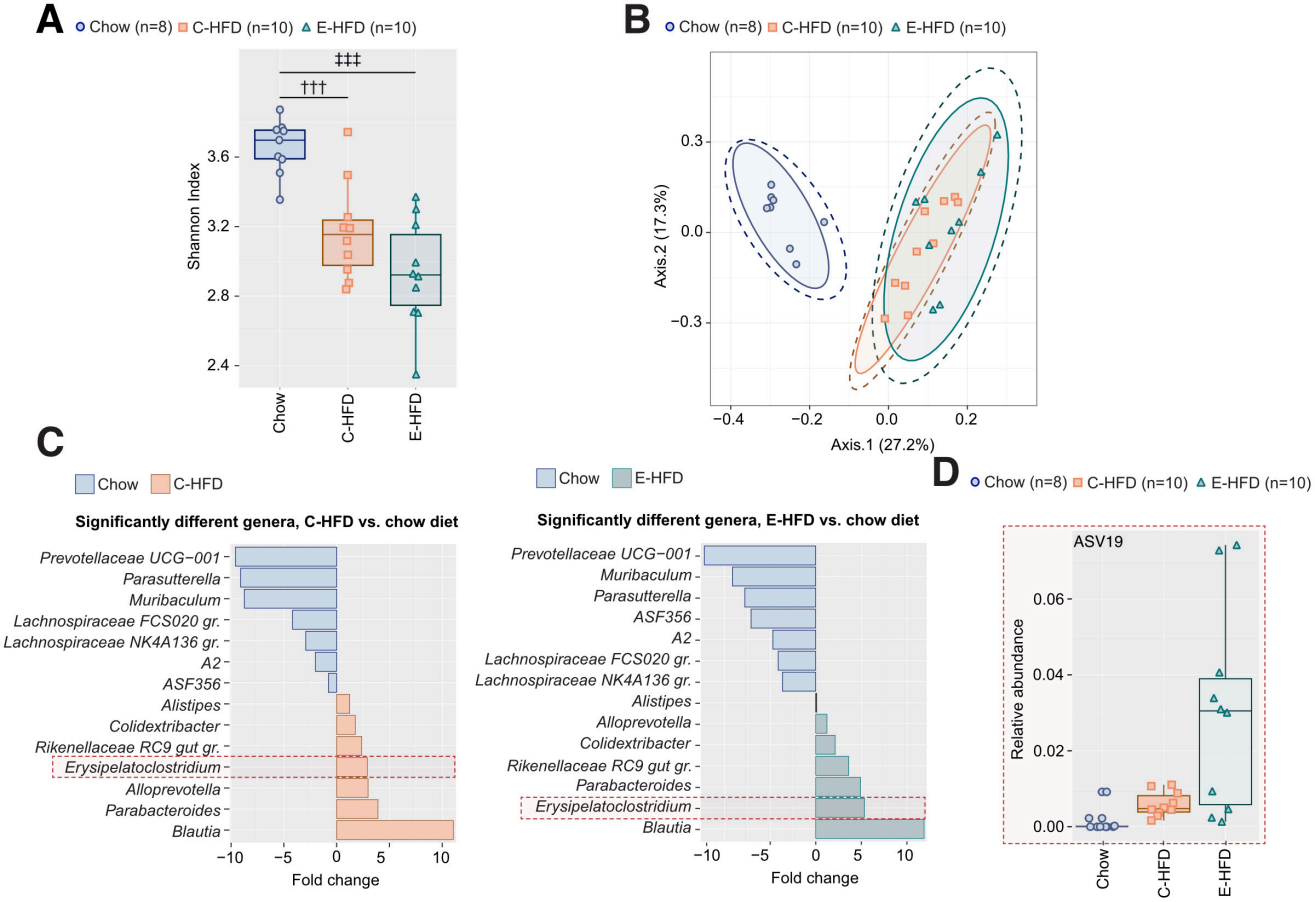
Decreased alpha diversity and relative abundance of given genera upon both HFDs. 5–7-week-old wt C57BL/6N male mice were fed either a control diet (blue circles), a C-HFD (orange squares), or an E-HFD (green triangles) for 1 week. **(A)** Shannon diversity index measured at the Assigned Sequence Variants (ASV) level. **(B)** Principal Component Analysis (PCA) plot based on the Bray-Curtis index on ASV levels. **(C)** Significant changes in the relative abundance of given genera in cecal content of E-HFD (left panel) and C-HFD (right panel). **(D)** Relative abundance of ASV19. Data represent four independent experiments, with each data point representing an individual mouse. Data are indicated as mean ± SEM; statistical significance was assessed using the Likelihood Ratio Test (LRT), with *p*-values corrected for multiple comparisons using the Benjamini-Hochberg method, and Wilcoxon rank sum test **(D)**; *p* = *p*-value. *E-HFD vs. C-HFD; ‡E-HFD vs. chow; †C-HFD vs. chow. *,‡,†*p* < 0.05; **,‡‡,††*p* < 0.01; ***,‡‡‡,†††*p* < 0.001.

Next, to investigate HFD-induced changes in the microbiota as a potential explanation for the distinct metabolic effects, we isolated DNA from the cecal content of E- and C-HFD-fed mice after 1 week and performed 16S rRNA gene amplicon sequencing. There was a significant decrease in alpha diversity upon treatment with either of the two HFDs compared to a control chow diet when using the Shannon diversity index, but no difference between the different HFDs (Figure 4A). In addition, a clear separation was visible on the first two axes of a Principal Coordinates Analysis (PCoA) plot based on the Bray-Curtis index on the assigned sequence variants (ASV) level separating the two HFD groups from the control mice on the chow diet (Figure 4B). This difference was also reflected in a differential abundance analysis where there were significant changes in the relative abundance of given genera upon treatment with E- or C-HFD compared to the control chow diet (Figure 4C). Notably, there was a decrease in genera from *Prevotellaceae, Lachnospiraceae, Parasuterella, A2, Clostridium ASF356*, and *Muribaculum*. Conversely, *Blautia, Erysipelatoclostridium, Colidextribacter, Parabacteroides*, and a genus of *Rickenellaceae* showed increases. Differential abundance analysis across all ASVs identified one genus, *Erysipelatoclostridium* (ASV19), as significantly different between the E-HFD and C-HFD group (FDR-adjusted *p* = 0.015). ASV19 abundance was 2.34-fold higher in the E-HFD group compared to C-HFD (Figure 4D). In contrast, comparison of relative abundances did not reach statistical significance (exact *p* = 0.075; Figure 4D). Thus, changes in gut microbiota induced by E- and C-HFD were very similar and did most likely not explain the starkly contrasting metabolic phenotypes.

### 3.5 Differential metabolic partitioning of different fat sources promotes increased energy storage in E-HFD fed mice

**Figure 5:**
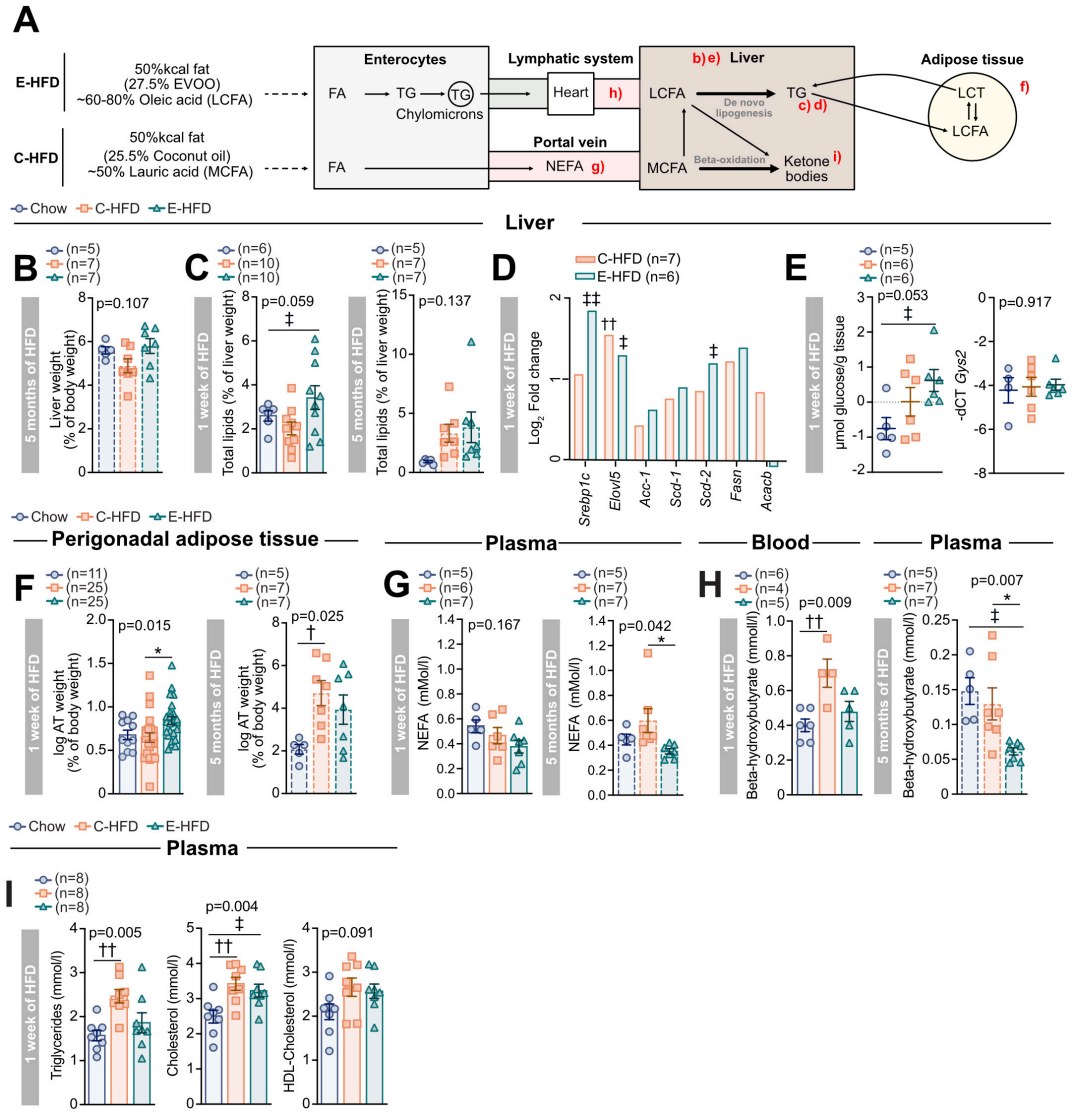
Differential metabolic partitioning of different fat sources promotes increased energy storage in E-HFD fed mice. 5–7-week-old wt C57BL/6N male mice were fed either a control diet (blue circles), a C-HFD (orange squares), or an E-HFD (green triangles) for 1 week to 5 months. **(A)** Schematic representation of the metabolic fate of dietary long-chain fatty acids (LCFAs) and medium-chain fatty acids (MCFAs). LCFAs, abundant in the E-HFD, are incorporated into chylomicrons, enter the lymphatic system, and reach the circulation via the left subclavian vein, facilitating peripheral deposition and hepatic uptake. In contrast, MCFAs, predominant in the C-HFD, bypass the lymphatic system and enter the portal vein directly for rapid hepatic metabolism. **(B)** Liver weight after 5 months of HFD feeding. **(C)** Total liver after 1 week and 5 months, and **(D)** expression of lipid metabolism genes (qPCR) after 1 week of HFD. **(E)** Hepatic glycogen content and expression of *Gys2* (glycogen synthase 2) after 1 week of HFD. **(F)** Perigonadal adipose tissue weight after and 5 months of HFD. **(G)** Plasma non-esterified fatty acids (NEFA) after 1 week and 5 months of HFD. **(H)** Plasma beta-hydroxybutyrate after 1 week and 5 months of HFD. **(I)** Plasma triglycerides, total cholesterol, and HDL-cholesterol after 1 week of HFD. Data represent five **(C–F)**, four **(H)**, and one **(B, G, I)** independent experiments, with each data point representing an individual mouse. *E-HFD vs. C-HFD; ‡E-HFD vs. chow; †C-HFD vs. chow. *,‡,†*p* < 0.05; **,‡‡,††*p* < 0.01; ***,‡‡‡,†††*p* < 0.001; Ordinary one-way ANOVA and multiple comparisons or unpaired Mann–Whitney *U*-test with two-tailed distribution. Data are Mean ± SEM; *p* = *p*-value.

Next, we examined whether differences in fatty acid composition and resulting metabolic partitioning could explain the distinct metabolic effects of the two HFDs. EVOO is primarily composed of oleic acid (OA; 60%−80% of total FA content), a long-chain fatty acid (LCFA) (38), whereas coconut oil predominantly consists of lauric acid (LA; ~50%), a medium-chain fatty acid (MCFA) (39). LCFAs are mainly transported via chylomicrons and preferentially stored in adipose tissue and liver, while MCFAs are transported directly via the portal vein into the liver, where they are metabolized through beta-oxidation to serve as an immediate energy source (Figure 5A) (40–42).

Liver weight between the two HFDs was comparable after 5 months of treatment (Figure 5B). However, total liver lipid content was significantly elevated in E-HFD-fed mice compared to control chow mice already after 1 week of dietary intervention, indicating enhanced lipid accumulation at this early timepoint (Figure 5C). This difference was no longer present at 5 months (Figure 5C). Moreover, gene expression analysis revealed an upregulation of key lipogenic genes, particularly *Srebp1c*, in the livers of E-HFD-fed mice, hence lipogenesis contributing to the increase in liver lipid content (Figure 5D). Liver glycogen content was also significantly increased in E-HFD mice, despite no changes in glycogen synthase 2 (*Gys2*) expression (Figure 5E), consistent with hyperglycemia driving insulin-independent glucose uptake into the liver. Furthermore, after 1 week of HFD feeding, perigonadal adipose tissue weight was significantly increased in E-HFD-fed mice compared to C-HFD-fed mice (Figure 5F). By 5 months, the effect reversed, with C-HFD-fed mice exhibiting significantly higher perigonadal adipose tissue weight than E-HFD-fed mice (Figure 5F).

Non-esterified fatty acids (NEFA) were higher in the plasma of mice fed a MCFA-rich C-HFD compared to E-HFD after 5 months, which was not yet significant after 1 week (Figure 5G). Consistent with energy utilization, the ketone body beta-hydroxybutyrate was significantly increased in C-HFD after 1 week of HFD compared to chow fed mice. Following 5 months of HFD, beta-hydroxybutyrate levels were significantly lower in E-HFD mice compared to both chow-fed and C-HFD mice (Figure 5H). Plasma triglycerides were increased in C-HFD-fed mice, whereas total cholesterol and HDL-cholesterol levels were similarly increased in both HFD-fed groups (Figure 5I).

Overall, these findings highlight differential metabolic partitioning: some phenotypes, such as early liver lipid accumulation and perigonadal adipose tissue weight in E-HFD-fed mice, were transient, whereas others, such as changes in beta-hydroxybutyrate levels, persisted over the 5-month dietary intervention.

## 4 Discussion

### 4.1 Key findings

Our study investigated the effects of an extra virgin olive oil-based HFD (EVOO, E-HFD) compared to a coconut oil-based HFD (C-HFD) in mice, focusing on glucose. Mice fed an E-HFD demonstrated significantly impaired glucose tolerance and blunted compensatory insulin secretion within 1 week when compared to mice fed a C-HFD despite unchanged body weight. Importantly, the insulinogenic index, a measure of insulin secretion, showed a decline over time, from 1 week to 5 months. Unaltered *ex vivo* glucose-stimulated insulin secretion in pancreatic islets, together with preserved islet morphology and beta-cell identity, indicated an islet-extrinsic factor that impairs beta-cell function *in vivo*.

### 4.2 Different fatty acids in plant-based HFDs

Oleic acid is the predominant fatty acid in extra virgin olive oil (EVOO). Its effects on islet cell function have been investigated in *in vitro* studies, yielding divergent results – ranging from attenuation to enhancement of insulin secretion in beta-cell lines and primary islets (43, 44). A key limitation of *in vitro* models is the use of pharmacological doses that may not reflect physiological conditions, as well as the lack of consideration for metabolites present *in vivo*. Previous *in vivo* studies have compared EVOO with lard-based HFDs and indicated beneficial effects of EVOO on glycemic control (8). However, it is important to note that control mice fed a lard-based HFD developed significantly higher body weights than those fed an E-HFD, introducing a potential bias in glycemic outcomes. Also, when mice fed a 60% lard-based HFD were compared with mice fed the same HFD supplemented with 20% of olive oil, body weight was significantly different between the groups, confounding results on glucose tolerance (45). Therefore, the control diet is an important denominator to consider as changes in body weight influence glycemic outcomes. Hence, a major strength of our study is the *in vivo* set-up with two plant-based HFDs (EVOO vs. coconut oil) containing distinct fatty acids of different chain lengths with no effect on body weight, thus ensuring the robustness of the glycemic phenotype.

### 4.3 Differential effects of HFD on inflammation and glycemic control

Adipose tissue inflammation is a key feature of metabolic disease, characterized by an increase in inflammatory adipose tissue macrophages (ATMs) and elevated levels of pro-inflammatory cytokines and chemokines (19). This local immune response is typically linked to systemic low-grade inflammation, marked by elevated plasma levels of pro-inflammatory cytokines such as IL-1β, IL-6, and TNF, which are associated with type 2 diabetes and metabolic disease (19, 46, 47). We have previously shown that the source of dietary fat influences the degree of gut inflammation as a lard-based HFD triggered a more pronounced inflammatory state in the gut compared to a C-HFD (20). EVOO, which is rich in phenolic compounds, has been shown to exhibit anti-inflammatory properties (4, 48, 49). For example, oleocanthal was reported to have similar properties to ibuprofen, acting as a natural anti-inflammatory agent by inhibiting cyclooxygenase (COX) enzymes, thereby reducing the production of pro-inflammatory mediators and attenuating inflammation (50). Clinical trials further support this anti-inflammatory effect, as diets rich in EVOO have been shown to decrease levels of inflammatory markers in the blood, suggesting reduced systemic inflammation (48). Indeed, we observed an uncoupeling between glycemic control and the inflammatory state upon EVOO. Mice fed an E-HFD, despite their poorer glycemic control, exhibited less adipose tissue and gut inflammation than mice fed a C-HFD. In addition, we found no differences in liver or systemic inflammation between HFD groups. These findings suggest that inflammation is unlikely to be the primary driver of impaired glucose tolerance in E-HFD fed mice, indicating an uncoupling of metabolic and immunological responses with EVOO.

### 4.4 Differential effects of HFD on microbiome

There is substantial evidence that the gut microbiome is altered in type 2 diabetes and obesity (51–54). The microbiome of HFD-fed mice may also contribute to the metabolic phenotype. Previous studies have suggested a beneficial impact of EVOO on gut microbiota. For example, Millman et al. (27) demonstrated a significant increase in alpha diversity in mice fed an E-HFD compared to those fed a lard-based HFD or a low-fat purified diet after a 10-week intervention. Similarly, another study reported increased beta diversity in mice consuming an EVOO-enriched diet compared to those on a lard-based HFD (55). However, these studies primarily compared EVOO to animal fat (lard)-based HFDs after prolonged intervention periods. In humans, EVOO intake has also been associated with microbiome alterations. For example, Luisi et al. examined the fecal microbiota of 18 overweight and obese participants and healthy controls following a Mediterranean diet enriched with EVOO for 3 months. They observed a notable increase in lactic acid bacteria compared to baseline levels (56). However, the Mediterranean diet is inherently rich in fiber, vegetables, and other bioactive compounds, making it difficult to attribute observed microbiome changes to a single dietary component. Moreover, while alterations in gut microbiota have been documented, this does not necessarily imply a direct impact on glycemic control. Indeed, in our study, we found no major differences between the E- and C-HFD microbiomes, with only one genus, *Erysipelatoclostridium*, differing between the two HFDs after 1 week. *Erysipelatoclostridium* belongs to the order *Erysipelotrichales*, which has been reported to be significantly decreased in mice that underwent one-anastomosis gastric bypass surgery and subsequently exhibited improved glucose metabolism (57). This raises the possibility that the increased genus of *Erysipelatoclostridium* could be involved in the observed glucose intolerance in E-HFD mice. Further work is needed to investigate a possible role for *Erysipelatoclostridium* in metabolic control in more detail.

### 4.5 Metabolic partitioning of different fatty acids

Lastly, we postulated that differences in fatty acid chain length could influence glucose homeostasis by affecting fatty acid tissue distribution and/or utilization across tissues, a process referred to as metabolic partitioning (58–61). In our study, E-HFD led to increased total liver lipids, *de novo* lipogenesis, and glycogen storage, along with greater perigonadal adipose tissue weight after just 1 week of HFD when compared to C-HFD. This adipose tissue expansion may be attributed to enhanced adipogenesis recently reported in response to dietary oleic acid intake (62). In addition, NEFAs and beta-hydroxybutyrate were higher in mice fed a C-HFD. This suggests that LCFAs from E-HFD are primarily directed toward energy storage, in line with previous studies (63–65), while MCFAs from C-HFD are used for energy utilization. Collectively, these findings suggest that the metabolic partitioning of E-HFD and C-HFD differs based on the chain length of the predominant fatty acid and occurs alongside impaired glucose tolerance, pointing to a link between fatty acid utilization and glycemic control.

### 4.6 Potential drivers for reduced insulin secretion

We can only speculate on the causal link between increased energy storage/ hepatic lipogenesis and dampened compensatory insulin secretion/ glucose intolerance upon exposure to LCFAs. Increased hepatic lipogenesis may alter the secretion of hepatokines, which are liver-derived proteins that can affect insulin sensitivity and secretion (66). Additionally, sphingolipids—particularly ceramides—may contribute to glucose intolerance in response to E-HFD, as ceramides are known to be affected by HFD and play a role in metabolic processes such as insulin signaling and inflammation (67, 68). Besides extrinsic factors such as plasma hepatokines or sphingolipids, also neuronal innervation could be involved, which may subsequently impact beta-cell function, leading to reduced insulin secretion *in vivo* (69, 70). Moreover, the saturated energy stores resulting from a LCFA diet could transiently impede glucose distribution during the GTT, thereby predominantly impairing glucose tolerance. The precise relationship between energy storage, hepatic lipogenesis, and reduced insulin secretion warrants further investigation.

### 4.7 Limitations

There are several limitations to this study that should be acknowledged. First, only male animals were included. This choice was done to align with the original study on which our experimental design was based (20). However, given the well-established sex-specific differences in metabolism and glucose homeostasis, the findings cannot be generalized to females. Future studies should therefore include both sexes to determine whether exposure to EVOO induces sex-dependent effects on metabolic partitioning. Second, the EVOO and coconut diet itself may introduce considerable variability. EVOO and coconut oil are natural products that exhibit batch-to-batch differences in fatty acid composition, polyphenol content, and other bioactive components. Future studies would benefit from a more detailed compositional characterization of the dietary oils, including fatty acid profiles and phenolic compounds, to improve reproducibility and enable more precise mechanistic interpretation of the observed metabolic effects. The precise fatty acid and polyphenol composition of the oils used in this study was not characterized, which limits our ability to attribute the observed effects to specific lipid species or bioactive compounds. Third, chow-fed control groups were not included in all functional analyses to contextualize the magnitude of dietary effects. For example, GSIS was not directly assessed side-by-side with the HFD conditions. This was mainly due to resource constraints. Fourth, baseline metabolic parameters were not systematically assessed in all experimental cohorts prior to dietary intervention. Hence, minor contributions of baseline variability cannot be fully excluded. Future studies should include systematic baseline metabolic assessments whenever feasible to ensure comparability between experimental groups before dietary interventions. Fifth, circulating inflammatory markers were not measured at later time points of the study due to limited availability of biological material from the long-term *in vivo* experiments. Given the important role of inflammation in metabolic disease and in the context of the present study, future investigations should include longitudinal measurements of inflammatory markers to better characterize the inflammatory contribution to the observed metabolic phenotypes. Lastly, based on our data, we propose a disrupted liver-pancreas axis. However, this remains speculative and lacks direct, mechanistic confirmation. In particular, comprehensive lipidomic profiling was not performed. Future studies should undertake such analyses to strengthen causal inference and further elucidate the molecular mechanisms linking enhanced energy storage under E-HFD to impaired glucose tolerance.

## 5 Conclusion

In conclusion, HFD based on EVOO has tissue-specific metabolic and immunological effects. While we found anti-inflammatory properties in perigonadal adipose tissue and the colon, EVOO led to adverse outcomes in the liver, as demonstrated by increased liver lipids, enhanced lipogenesis, and glycogenesis, and dampened compensatory insulin secretion, resulting in pronounced glucose intolerance. This underscores the need to carefully consider dietary fat types based on chain length and dosages in nutritional strategies to manage metabolic health. Further research is essential to define the conditions under which EVOO might be beneficial vs. harmful and to elucidate the underlying mechanisms, thereby aligning our findings with broader dietary guidelines.

## Data Availability

The data presented in the study are deposited in the NCBI Sequence Read Archive (SRA), accession number PRJNA1441538 (https://www.ncbi.nlm.nih.gov/sra/PRJNA1441538).
