# Supplementary material for: An extra virgin olive oil diet impairs glycemic control, but mitigates high-fat diet-induced inflammation compared to coconut oil in mice
Source: Front Nutr. 2026 Apr 28;13:1776312. doi: 10.3389/fnut.2026.1776312 (PMC13163686; doi:10.3389/fnut.2026.1776312)
Supplement: Supplementary file 1 [file Data_Sheet_1.pdf]

Supplementary Material

1 Methods

1.1 Animal model and ethical considerations

The C57BL/6N mouse model was chosen for its relevance to human biology, particularly in studying type 2 diabetes and obesity, as it closely mimics the initial phase of the disease characterized by impaired glucose tolerance and insulin resistance [1]. To minimize pain, suffering, and distress, mice were group-housed and subjected to minor husbandry changes, such as dietary modifications. Blood samples for glucose and insulin measurements were obtained via tail vein punctures, ensuring minimal discomfort. Adverse events were not expected beyond minor weight changes, which were closely monitored. Humane endpoints were established, including criteria for severe weight loss (>15%), behavioral changes, or signs of distress, with regular monitoring and a cumulative scoring system to determine the need for euthanasia.

1.2 Metabolic *in vivo* assessments

Metabolic state was assessed via intraperitoneal (i.p.) glucose (GTT), insulin (ITT), and pyruvate tolerance tests (PTT). GTTs were conducted after 1 week of HFD or on a monthly basis. Mice were fasted 6 h, then injected i.p. with glucose (2 g/kg body weight, Braun, Germany), and blood glucose was measured at baseline and up to 120 min post-injection using a glucometer (FreeStyle, Abbott, USA) with blood collected via tail vein puncture. For plasma insulin measurements, blood samples were collected at the first 3 time points using hematocrit capillaries. The collected blood was transferred into 1.5 mL tubes containing 3 µL 50 mM EDTA, spun down (12290 g, 5 min, 4°C), and stored at -20 °C upon analysis. Insulin levels were quantified by electrochemiluminescence (MESO SECTOR S 600, MSD, USA). ITTs and PTTs were performed after 1 week or 5 months of HFD, with 3 h fasted mice injected with insulin (1 U/kg body weight, Actrapid Penfill Insulin 100 IU/ml, Novo Nordisk) or pyruvate (10 µl/g of 20% pyruvate solution in 0.9% NaCl, pH 7.4, Sigma-Aldrich, USA) respectively.

1.3 Immune cell isolation

Colon: Colons were cleaned and sectioned, and epithelial cells were removed by shaking in HBSS with 2mM EDTA (Gibco, USA). Tissues were digested in a collagenase VIII mix (Complete (10% FBS, P/S, Glutamax; Gibco, USA) Iscove's Modified Dulbecco's Medium (IMDM, Sigma-Aldrich, USA), 2 mg/mL collagenase VIII (#C2139; Sigma-Aldrich, USA), 25 µg/mL DNase I (#11284932001, Roche, Switzerland)) for 30 min at 37°C, homogenized using a gentleMACS Octo Dissociator (Militenyi Biotec; program: ms_intestine-01), filtered (70 µm (#130-098-462, Militenyi Biotec, Germany)), and leukocytes enriched via a percoll gradient (40%/70%; #GE17-0891-01; GE Healthcare, USA). Recovered immune cells, were washed and strained through a 35 µm strainer FACS tube (#352235, Corning, USA).

Adipose tissue: Perigonadal fat pads were minced and digested with collagenase IV (#C2139; Worthington, USA) mix (1x HBSS, 10m M HEPES (Gibco, USA), 1.5 mg/ml collagenase IV and 8.25 µg/ml DNAse I (#11284932001, Roche, Switzerland)) at 400 rpm for 20 min at 37°C. Digestion was

stopped with FACS buffer (1xDPBS, 0.5% BSA, 5 mM EDTA), followed by filtration through a cotton gauze, erythrocyte lysis with Red Cell Lysis Buffer (154 mM NH4Cl, 10 mM KHCO3, 0.1 mM EDTA), and straining through 35 µm strainer FACS tube (#352235, Corning, USA).

Liver: Livers were cut into small pieces, digested with collagenase IV mix (#C2139; Worthington, USA) mix (1x HBSS, 10m M HEPES (Gibco, USA), 1.5 mg/ml collagenase IV and 8.25 µg/ml DNAse I (#11284932001, Roche, Switzerland)) for 30 min at 37°C. Samples were vortexed before starting the digestion and 15 min later, as well as before filtering through a 70 µm sieve (#130-098-462, Militenyi Biotec, Germany). Immune cells were enriched using a percoll gradient as described above (colon).

1.4 Flow cytometry of immune cells

Cells were pelleted, Fc receptor was blocked using CD16/32, and the corresponding antibody staining mix (**Supplementary Table 2**) was added and incubated for 30 min in the dark on ice. As a final step, cells were washed and resuspended in FACS buffer (1xDPBS, 0.5% BSA, 5 mM EDTA) before acquiring with a BD LSR II Fortessa (BD, USA). Data analysis was performed using FlowJo software 10.8.2 (BD, USA) and following gating strategies (**Supplementary Figures 1-3**).

1.5 Gating strategies

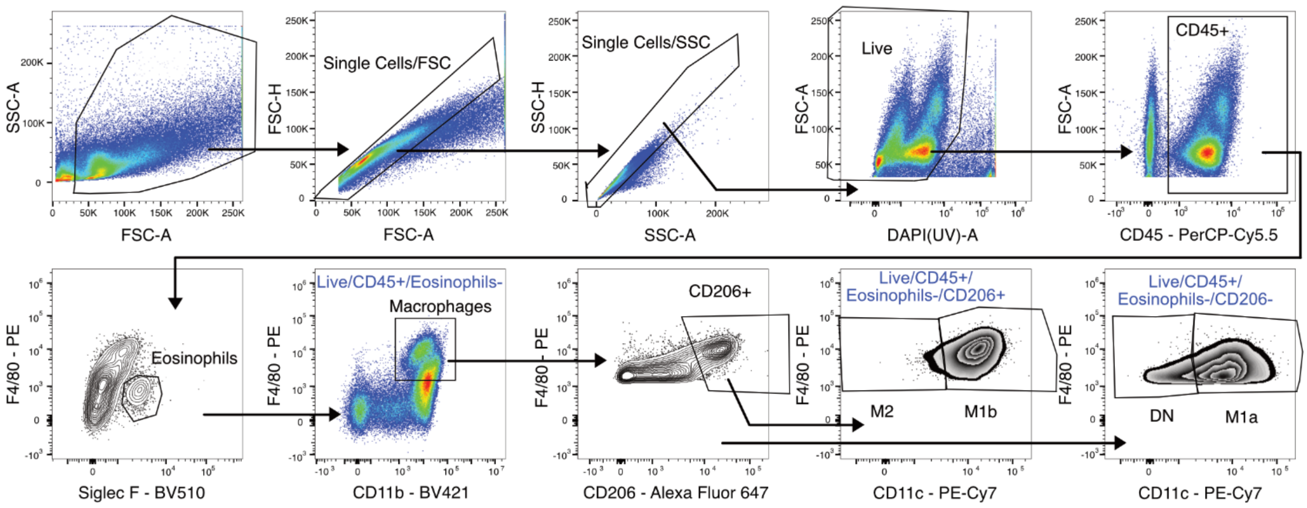

Supplementary Figure 1: **Gating strategy for flow cytometry analysis of adipose tissue macrophages.** Representative gating of enriched Lymphocytes/Single cells/Live/CD45$^+$ (used for all following flow cytometry analysis). Eosinophils were identified as Siglec F$^+$ and F4/80$^-$. Macrophages were analyzed excluding eosinophils and gated as CD11b$^+$ and F4/80$^+$. Subclassification of macrophages included M2 (CD206$^+$ and CD11c$^+$) and M1b (CD206$^+$ and CD11c$^-$). CD206$^-$ macrophages were further identified as M1a (CD11c$^+$) or as double negative (DN) macrophages (CD11c$^-$).

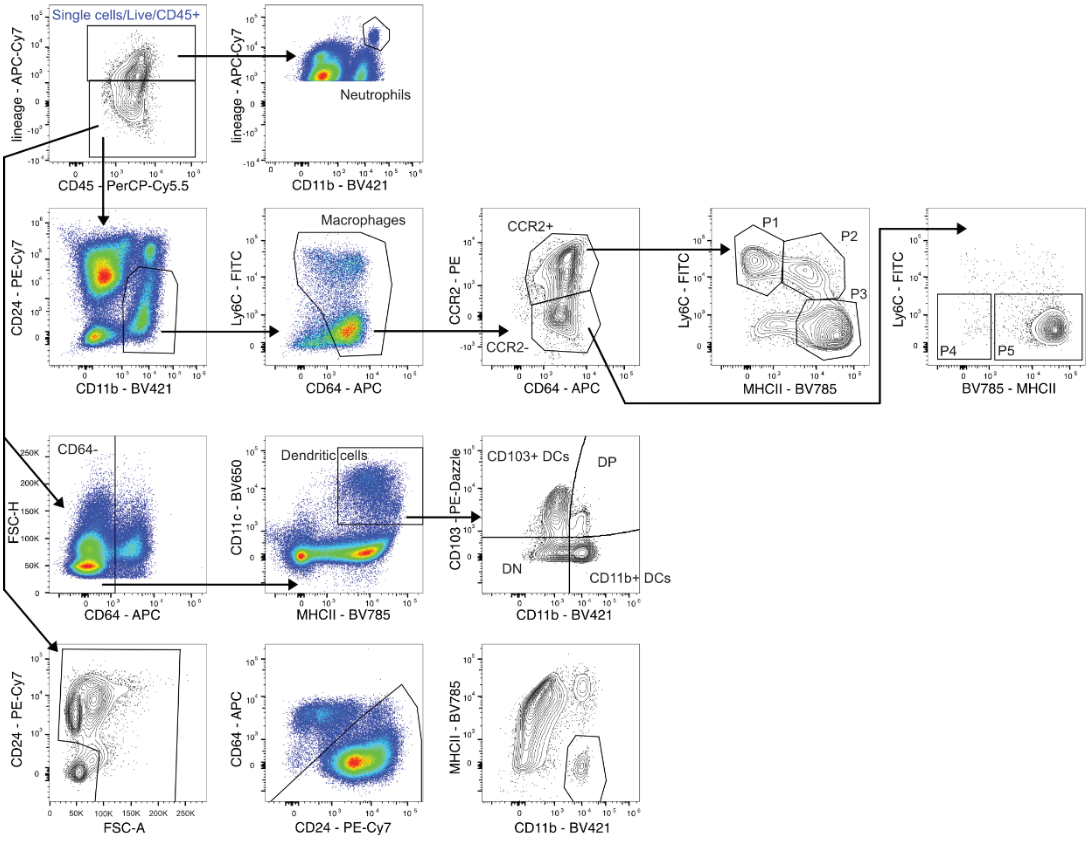

Supplementary Figure 2: Gating strategy for flow cytometry analysis of colonic innate immune cells. Representative flow cytometry plot identified intestinal neutrophils ($CD45^{+}Lin^{+}/CD11b^{high}$), intestinal macrophage subpopulations: pro-inflammatory $CCR2^{+}$ were delineated into P1, P2, and intermediate P3 subpopulations ($Lye6C^{+}MHCII^{+}$), while anti-inflammatory/resident $CCR2^{-}$ were divided into P4 and P5 subpopulations ($Ly6C^{-}MHCII^{+}$). Furthermore, cells were gated as $CD45^{+}Lin^{-}/CD64^{-}/MHCII^{+}/CD11c^{+}$ for dendritic cells (DCs), with additional gating for $CD103^{+}$, $CD11b^{+}$, $CD103^{+}/CD11b^{+}$, and double negative (DN) dendritic cell subpopulations. Eosinophils were gated as $CD45^{+}Lin^{-}$ $/CD24^{+}/CD64^{-}/CD11b^{+}/MHCII^{-}$.

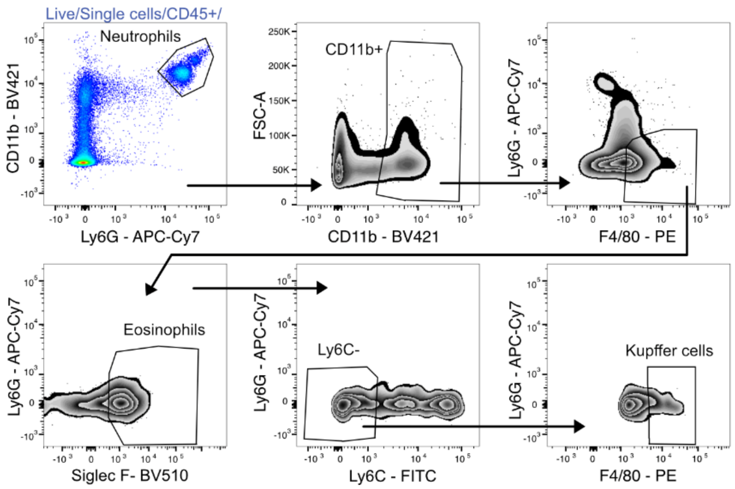

Supplementary Figure 3: Gating strategy for flow cytometry analysis of liver innate immune cells.
Neutrophils were identified as $CD11b^{+}/Ly6G^{+}$. After excluding neutrophils, further gating was performed on $CD11b^{+}/F4/80^{+}/Siglec\ F^{+}$ to identify eosinophils. Cells not captured in these gates were gated for $Ly6C^{-}$ and $F4/80^{+}$ to specifically identify Kupffer cells, the resident liver macrophages.

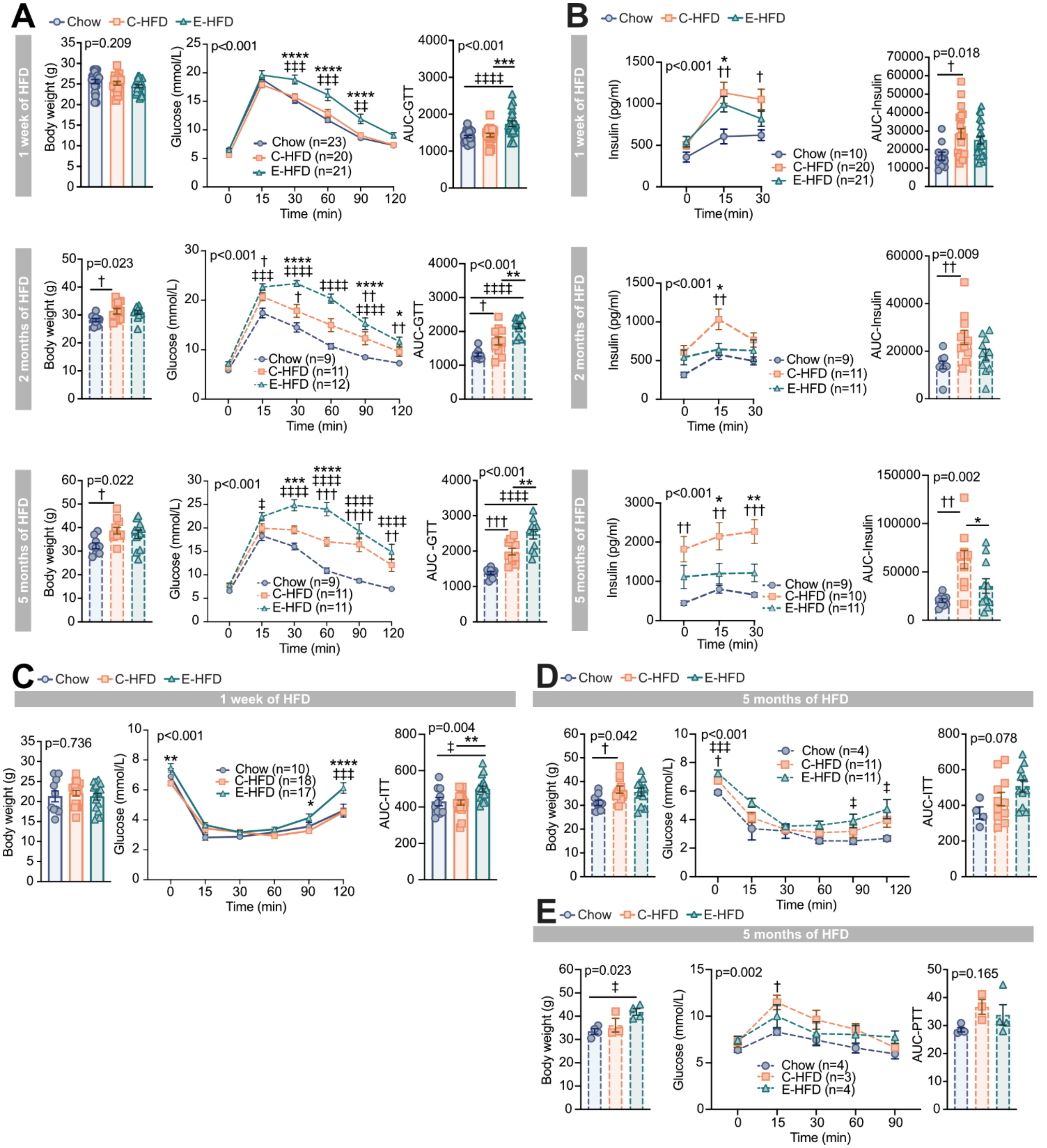

Supplementary Figure 4: E-HFD leads to pronounced glucose intolerance due to reduced insulin secretion (extension of main Figure 1 including chow control). 5-7-week-old wild-type C57BL/6N male mice were fed either a control diet (chow, blue circles), a coconut-based HFD (orange squares), or an extra-virgin olive oil-based HFD (green triangles) for 1 week for up to 5 months. (**A**) Body weight, intraperitoneal glucose tolerance test (GTT), and area under the curve (AUC) after 1 week, 2 months, and 5 months of HFD. (**B**) Insulin values for the first 30 min of the GTT and AUC, respectively. (**C**) Body weight, intraperitoneal insulin tolerance test (ITT), and AUC after 1 week and (**D**) 5 months of HFD feeding compared to chow. (**E**) Body weight, intraperitoneal pyruvate tolerance test (PTT), and AUC after 5 months of HFD. Data represent five (A, B: for 1 week of HFD), two (A, B: for 2 and 5 months of HFD, C, D), and one (E) independent experiments, with each data point representing an individual mouse. * E-HFD vs C-HFD; # E-HFD vs chow; † C-HFD vs

chow. *, ‡, †, & < 0.05; **, ‡ ‡, †† p < 0.01; ***, ‡ ‡ ‡, ††† p < 0.001; 2-way ANOVA, and multiple comparison or Ordinary one-way ANOVA and multiple comparisons. Data are Mean +/- SEM; p=p-value.

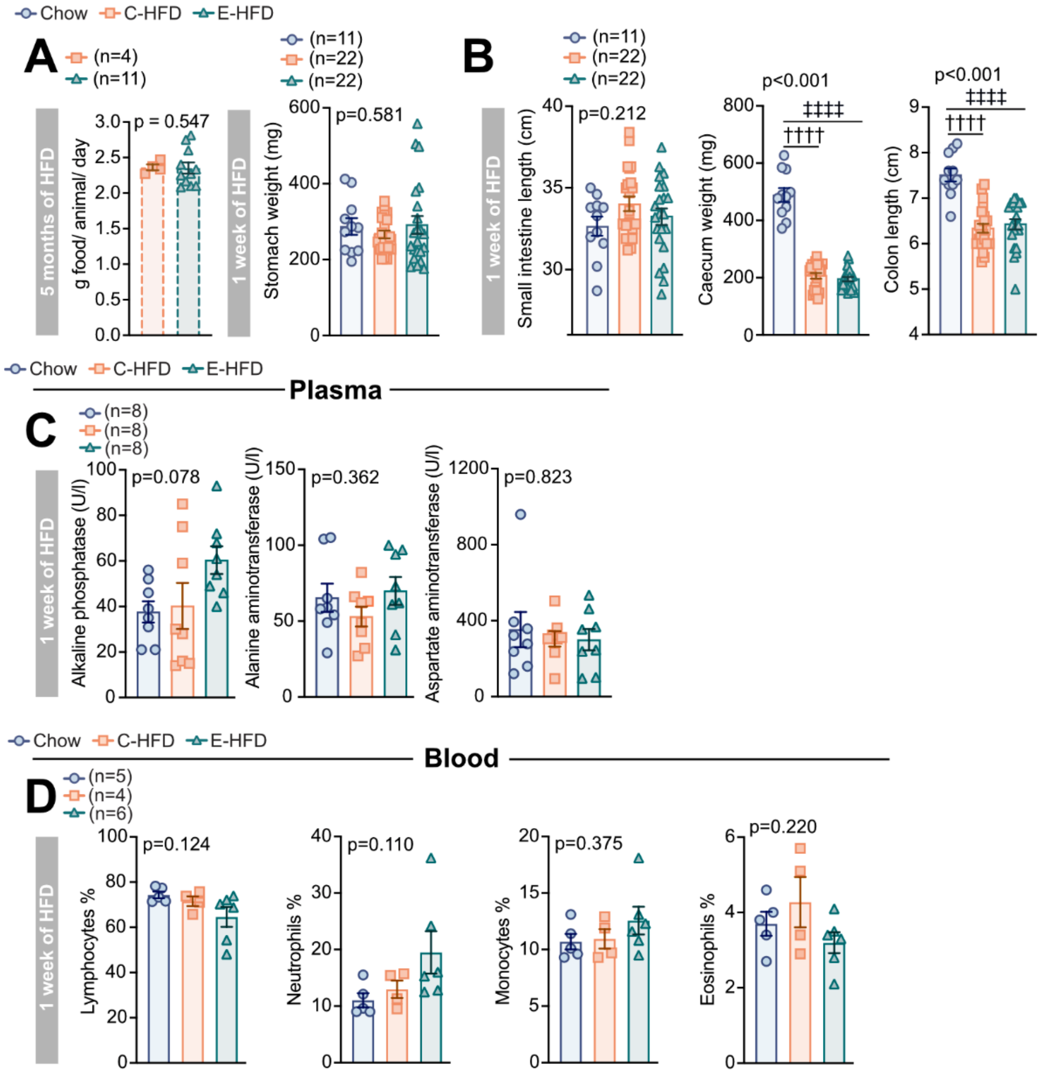

Supplementary Figure 5: Food intake, macroscopic features, liver enzymes, and blood immune cells in response to C- or E-HFD. 5-7-week-old wild-type C57BL/6N male mice were fed either a control diet (chow, blue circles), a coconut-based HFD (orange squares), or an extra-virgin olive oil-based HFD (green triangles) for 1 week. (**A**) Food intake and stomach weight. (**B**) Small intestine length, cecum weight, and colon length. (**C**) Plasma concentrations of liver enzymes, and (**D**) blood analysis of immune cells after 1 week of HFD. Data represent five (A: stomach weight; B), four (C, A: food intake), and one (D) independent experiments, with each data point representing an individual mouse * E-HFD vs C-HFD; ‡ E-HFD vs chow; † C-HFD vs chow. *, ‡, †, & < 0.05; **, ‡ ‡, †† p < 0.01; ***, ‡ ‡ ‡, ††† p < 0.001; Ordinary one-way ANOVA and multiple comparisons or unpaired Mann–Whitney U test with two-tailed distribution. Data are Mean +/- SEM; p=p-value.

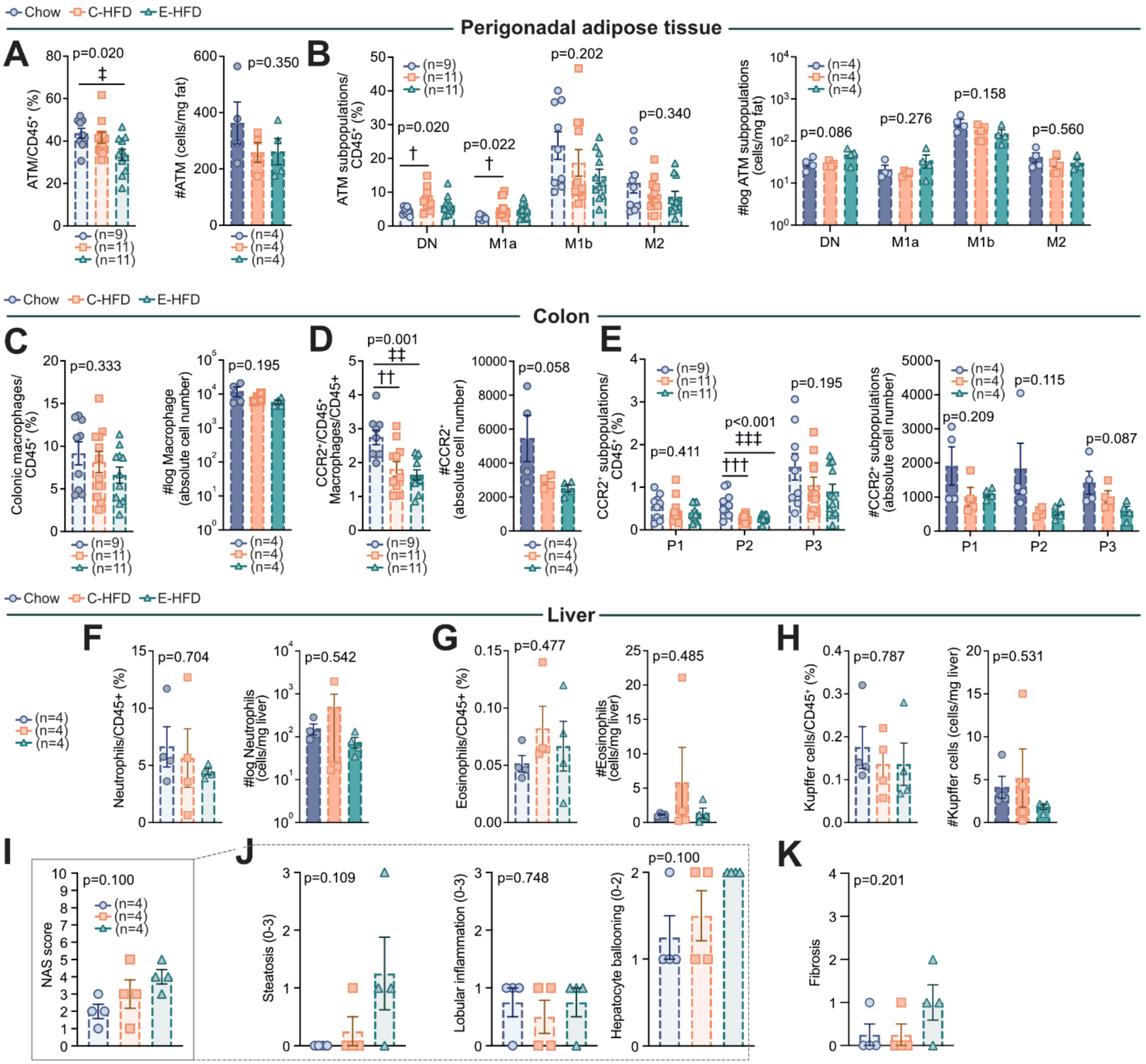

Supplementary Figure 6: C-HFD, but not E-HFD, causes persistent M1a-driven adipose tissue inflammation after 5 months of HFD. 5 to 7-week-old wt C57BL/6N male mice were fed either a control chow diet (blue circles), a C-HFD (orange squares), or an E-HFD (green triangles) for 5 months. (**A**) Analysis of flow cytometry frequencies of perigonadal adipose tissue macrophages (ATMs) and absolute cell numbers. (**B**) Analysis of flow cytometry frequencies of ATM subpopulations and absolute cell numbers. (**c**) Analysis of flow cytometry frequencies of colon macrophages and absolute cell numbers; and (**D**) colon $CCR2^+$ macrophages and absolute cell numbers. (**E**) Analysis of flow cytometry frequencies of colon $CCR2^+$ macrophage subpopulations (P1-P3) and absolute cell numbers. (**F**) Analysis of flow cytometry frequencies of liver neutrophils, (**G**) liver eosinophils, (**H**) and liver Kupffer cells and absolute cell numbers, respectively. (**I**) Non-Alcoholic Steatohepatitis (NAS) score and underlying analysis of (**J**) steatosis, lobular inflammation, and hepatocyte ballooning. (**K**) Liver fibrosis score. Absolute cell numbers are shown in darker shades of the bars. Data represent tow (A-E for frequencies), and one (A-E: for absolute cell numbers, F-K) independent experiments, with each data point representing an individual mouse. * E-HFD vs C-HFD; ‡ E-HFD vs chow; † C-HFD vs chow. *, ‡, † p<0.05; **, ‡‡, †† p<0.01; ***, ‡‡‡, ††† p<0.001; Ordinary one-way ANOVA and multiple comparison; Data are Mean +/- SEM; p=p-value.

2 Reagents and resources

	Chow diet	Coconut oil-based HFD (C-HFD)	Extra virgin olive oil-based HFD (E-HFD)
**Crude Fat (%)**	4.5	27.6 (50 kcal%: 27.5% extra virgin olive oil)	27.6 (50 kcal%:2% soybean oil, 25.5% coconut oil)
**Crude Protein (%)**	18.5	20.2	20.2
**Crude Fiber (%)**	4.5	1.8	1.8
**Crude ash (%)**	6.5	5	5
**Sugar (%)**	0	22.3	22.3
**Starch (%)**	35	0.1	0.1
**Additives per kg**			
**Vitamin A (IE/IU)**	18000	15000	15000
**Vitamin D3 (IE/IU)**	1000	1500	1500
**Vitamin E (mg)**	132	150	150
**Vitamin C (mg)**	68	30	30
**Copper (mg)**	14	12	12

Table 1: Diet composition of control diets and HFDs

Gene	Forward Primer	Reverse Primer
**Ppia**	5′-GAGCTGTTTGCAGACAAAGTTC-3′	5′-CCCTGGCACATGAATCCTGG-3′
**B2m**	5′-TTCTGGTGCTTGTCTCACTGA-3′	5′-CAGTATGTTCGGCTTCCCATTC-3′
**Gapdh**	5′-AGGTCGGTGTGAACGGATTTG-3′	5′-TGTAGACCATGTAGTTGAGGTCA-3′
**Ins2**	5′-TGGCTTCTTCTACACACCCAAG-3′	5′-ACAATGCCACGCTTCTGCC-3′
**Pdx1**	5′-CCCCAGTTTACAAGCTCGCT-3′	5′-CTCGGTTCCATTCGGGAAAGG-3′
**Pcsk1**	5′-ACGAGACTCCTGACGTGGA-3′	5′-GCACCTCGGGACCCAAATC-3′
**Xbp1s**	5′-TGAGTCCGCAGCAGGTG-3′	5′-AGATGTTCTGGGGAGGTGAC-3′
**Gcg**	5′-TTACTTTGTGGCTGGATTGCTT-3′	5′-AGTGGCGTTTGTCTTCATTCA-3′
**Srebp 1c**	5′-GGAGCCATGGATTGCACATT-3′	5′-GGCCCGGGAAGTCACTGT-3′
**Elovl5**	5′-CTGAGTGACGCATCGAAATG-3′	5′-CTTGCACATCCTCCTGCTC-3′
**Acc-1**	5′-CCTCCGTCAGCTCAGATACA-3′	5′-TTTACTAGGTGCAAGCCAGACA-3′
**Scd-1**	5′-CTGTACGGGATCATACTGGTTC-3′	5′-GCCGTGCCTTGTAAGTTCTG-3′
**Scd-2**	5′-TGCCTTGTATGTTCTGTGGC-3′	5′-TCCTGCAAGCTCTACACCTG-3′
**Fasn**	5′-AGCGGCCATTTCCATTGCCC-3′	5′-CCATGCCCAGAGGGTGGTTG-3′
**Acacb**	5′-CCCAGGAGGCTGCATTGA-3′	5′-AGACATGCTGGGCCTCATAGTA-3′
**Gys2**	5′-ACCAAGGCCAAAACGACAG-3′	5′-GGGCTCACATTGTTCTACTTGA-3′

Table 2: Primer sequences used for quantitative real time-PCR

Reagents and resources	Source	Identifier
Anti-mouse CD16/32 (93); Dilution (1:100)	Biolegend	Cat#101321;<br>RRID: AB_2103871
Anti-mouse CD11c (N418) BV650; Dilution (1:100)	Biolegend	Cat#117339;<br>RRID: AB_2562414
Anti-mouse CD11c (N418) PE-Cy7; Dilution (1:120)	Biolegend	Cat#117318;<br>RRID: AB_493568
Anti-mouse CD11b (M1/70) BV421; Dilution (1:40)	Biolegend	Cat#101236;<br>RRID: AB_11203704
Anti-mouse CD45 (30-F11) PerCP-Cy5.5; Dilution (1:300)	Biolegend	Cat#103131;<br>RRID: AB_893344
Anti-mouse I-A/I-E (M5/114.15.2) BV785; Dilution (1:500)	Biolegend	Cat#107645;<br>RRID: AB_2565977
Anti-mouse Ly6C (HK1.4) FITC; Dilution (1:350)	Biolegend	Cat#128005;<br>RRID: AB_1186134
Anti-mouse CCR2 (475301) PE; Dilution (1:25)	R&D Systems	Cat#FAB5538P;<br>RRID:AB_10718414
Anti-mouse CD103 (2E7) PE-Dazzle594; Dilution (1:80)	Biolegend	Cat#121430;<br>RRID: AB_2566493
Anti-mouse CD24 (M1/69) PE-Cy7; Dilution (1:160)	Biolegend	Cat#101821;<br>RRID: AB_756047
Anti-mouse CD64 (X54-5/7.1) APC; Dilution (1:60)	Biolegend	Cat#139306;<br>RRID: AB_11219391
Anti-mouse CD3 (145-2C11) APC-Cy7; Dilution (1:35)	Biolegend	Cat#100330;<br>RRID: AB_1877170;
Anti-mouse Nk1.1 (PK136) APC-Cy7; Dilution (1:40)	Biolegend	Cat#108723;<br>RRID: AB_830870
Anti-mouse CD19 (6D5) APC-Cy7; Dilution (1:600)	Biolegend	Cat#115530;<br>RRID: AB_830707
Anti-mouse Siglec F (E50-2440) BV510; Dilution (1:60)	BD Biosciences	Cat#740158;<br>RRID: AB_2739911
Anti-mouse F4/80 (BM8) PE; Dilution (1:100)	Biolegend	Cat#123110;<br>RRID: AB_893486
Anti-mouse CD206 (C068C2) A647; Dilution (1:160)	Biolegend	Cat#141712;<br>RRID: AB_10900420

Table 3: Antibodies

Assay	Manufacturer	Catalog Number
Mouse/Rat Insulin Kit	MesoScale Diagnostics	Cat#K152BZC
V-PLEX Proinflammatory Panel 1 Mouse Kit	MesoScale Diagnostics	Cat#K15048D-1

Table 4: Critical Commercial Assays

Software	Developer	Version	Website

FlowJo	Becton Dickinson & Company (BD)	Version 9.9 or higher	https://flowjo.com
BD FACSDiva	Becton Dickinson & Company (BD)	Version 8.0.1 or higher	https://www.bdbiosciences.com/en-us/instruments/research-instruments/research-software/flow-cytometry-acquisition/facsdiva-software
Prism	GraphPad Software, LLC	Version 10	https://www.graphpad.com
R	The R Foundation for Statistical Computing	Version 3.6 or higher	https://www.r-project.org

Table 5: Software

3 References

[1] Wang CY, Liao JK. A Mouse Model of Diet-Induced Obesity and Insulin Resistance. Methods Mol Biol 2012;821:421. https://doi.org/10.1007/978-1-61779-430-8_27.
